# Pharmaceuticals Removal by Adsorption with Montmorillonite Nanoclay

**DOI:** 10.3390/ijms22189670

**Published:** 2021-09-07

**Authors:** Marina Kryuchkova, Svetlana Batasheva, Farida Akhatova, Vasily Babaev, Daina Buzyurova, Anna Vikulina, Dmitry Volodkin, Rawil Fakhrullin, Elvira Rozhina

**Affiliations:** 1 Institute of Fundamental Medicine and Biology, Kazan Federal University, Kreml uramı 18, 420008 Kazan, Republic of Tatarstan, Russia; maricshka80@gmail.com (M.K.); svbatasheva@gmail.com (S.B.); akhatovaf@gmail.com (F.A.); 2 Arbuzov Institute of Organic and Physical Chemistry, FRC Kazan Scientific Center of RAS, Arbuzov St., 8, 420088 Kazan, Republic of Tatarstan, Russia; babaev@iopc.ru (V.B.); dainaibrasheva@gmail.com (D.B.); 3 Bavarian Polymer Institute, Friedrich-Alexander-University Erlangen-Nürenberg (FAU), Dr.-Mack-Straße 77, 90762 Fürth, Germany; anna.vikulina@fau.de; 4 Institute of Polymer Technology, Friedrich-Alexander-University Erlangen-Nürenberg (FAU), Dr.-Mack-Straße 77, 90762 Fürth, Germany; 5 School of Science and Technology, Nottingham Trent University, Clifton Lane, Nottingham NG11 8NS, UK; dmitry.volodkin@ntu.ac.uk

**Keywords:** pharmaceuticals, clay minerals, adsorption, treatment effectiveness

## Abstract

The problem of purifying domestic and hospital wastewater from pharmaceutical compounds is becoming more and more urgent every year, because of the continuous accumulation of chemical pollutants in the environment and the limited availability of freshwater resources. Clay adsorbents have been repeatedly proposed as adsorbents for treatment purposes, but natural clays are hydrophilic and can be inefficient for catching hydrophobic pharmaceuticals. In this paper, a comparison of adsorption properties of pristine montmorillonite (MMT) and montmorillonite modified with stearyl trimethyl ammonium (hydrophobic MMT-STA) towards carbamazepine, ibuprofen, and paracetamol pharmaceuticals was performed. The efficiency of adsorption was investigated under varying solution pH, temperature, contact time, initial concentration of pharmaceuticals, and adsorbate/adsorbent mass ratio. MMT-STA was better than pristine MMT at removing all the pharmaceuticals studied. The adsorption capacity of hydrophobic montmorillonite to pharmaceuticals decreased in the following order: carbamazepine (97%) > ibuprofen (95%) > paracetamol (63–67%). Adsorption isotherms were best described by Freundlich model. Within the pharmaceutical concentration range of 10–50 µg/mL, the most optimal mass ratio of adsorbates to adsorbents was 1:300, pH 6, and a temperature of 25 °C. Thus, MMT-STA could be used as an efficient adsorbent for deconta×ating water of carbamazepine, ibuprofen, and paracetamol.

## 1. Introduction

Nowadays, pharmaceuticals and their metabolites are increasingly found in the environments, accumulating in surface- and ground waters, and provoking toxicological effects on organisms in aquatic or terrestrial ecosystems [1]. There are many ways in which drugs can enter surface waters, with the prevailing sources of the drugs being wastewaters from pharmaceutical companies, municipal wastewater treatment plants, and hospitals [2,3]. To prevent the entry of pharmaceuticals into surface and ground waters disposal of drugs from various sources (industrial, hospital, domestic, agricultural wastewater) should be carefully controlled [4]. Detailed studies of the interaction between surface and groundwater must also be conducted, using new approaches and methods [5]. Improved analytical methods are needed for the detection of small amounts of pharmaceutical compounds in wastewater treatment plants [6,7]. Advanced drug-delivery systems capable of targeted delivery and controlled release of active agents can significantly reduce doses of applied medicines [8]. In the process of monitoring treatment facilities in a number of European countries, it was found that wastewater after treatment contained a wide range of drugs (carbamazepine, diclofenac, ibuprofen, ketoprofen, acetaminophen, etc.) [9,10]. For example, carbamazepine, an antiepileptic drug, is one of the most frequently reported pharmaceutical compounds in surface waters [10]. It was found in wastewaters in a number of countries, and the efficiency of biological treatment systems towards carbamazepine does not exceed 54% [11]. During the photolytic decomposition of carbamazepine, acridine derivatives are formed, which can cause chromosomal mutations [12], creating a risk of genetic changes in water inhabitants.

Paracetamol and ibuprofen are over-the-counter drugs that are widely used around the world and are often found in aquatic environments as a consequence of the low efficiency of water purification processes [13]. It has been shown that the presence of ibuprofen in the aquatic environment affects the reproduction of crustaceans *Daphnia magna* at a concentration of 13.4 mg/L [14] and paracetamol has a toxic effect on *D. magna* at concentrations from 9.2 to 50 mg/L [15].

Various methods are used for removing pharmaceuticals from water and wastewater, such as activated sludge systems [16,17], membrane bioreactors [18,19], electrocoagulation [20], magnetic ion-exchange resins [21], oxidation processes [22,23], and adsorption, the latter being the most effective and inexpensive method of drug removal [24,25]. One of the most popular adsorbents is activated carbon, but its relatively high cost and complex regeneration process prompt the search for alternative inexpensive adsorbents [26]. To ease the recovery of used adsorbents, they can be made responsive to an applied magnetic field, as it was successfully demonstrated with carbon nanotubes impregnated with a metal phase [27]. Adsorbents based on clay minerals [28] have also been considered as readily available natural materials that can be used to remove organic micropollutants [29,30,31].

The development of efficient water treatment systems is impeded by the high chemical diversity of pharmaceutical micropollutants, implying that no universal adsorbent can fit all the tasks. While natural clay adsorbents are hydrophilic, some of their target micropollutants are hydrophobic. Therefore, the hydrophobisation of the clay adsorbent can be used as way to increase the adsorption of hydrophobic micropollutants.

Montmorillonite (MMT) is a layered aluminum silicate with a general formula of (Na,Ca)_0.33_(Al,Mg)_2_(Si_4_O_10_)(OH)_2_· nH_2_O. The isomorphous substitutions of Si^4+^ by Al^3+^ in the tetrahedral sheet and Al^3+^ by Mg^2+^ in the octahedral ones imparts a negative charge to the clay, which is compensated by cations in the interlayer space [32]. The layered structure of MMT allows the adsorption of various substances both on the surfaces and in the interlayer spaces, and other MMT-related adsorbents with desired properties can be obtained by chemical modifications of the natural clay. The interlayer space makes up about 90% of the entire clay surface and enables MMT to absorb water molecules and other compounds [33]. High cation-exchange capacity of MMT is beneficial for its application as a drug carrier [34] or a sorbent [35]. MMT is widely used as an absorbent to remove hydrophilic cationic contaminants [36]. Due to the large amount of hydrated cations in the interlayer space, MMT has almost no adsorption capacity for anionic and hydrophobic organic pollutants. However, hydrated cations can be replaced by organic ammonium cations, after which the adsorption capacity for organic pollutants increases significantly [37]. When MMT is treated with a surfactant (for example, trimethyl stearyl ammonium), inorganic cations in the MMT are replaced by organic cations, which contribute to the appearance of hydrophobic properties and an increase in interlayer spacing, while the ordered layered structure of the particles is preserved.

Hydrophobic organoclay adsorbents for pharmaceuticals were obtained by MMT modification with octadecylamine [38], didodecyldimethylammonium bromide [39] benzyldimethyltetradecyl ammonium (BDTA), and hexadecyltrimethylammonium (HDTMA) [40,41]. So far, the sorption properties of hydrophobic MMT were tested for a limited number of pharmaceuticals including carbamazapine [39,40,41] and ibuprofen [38,39,40], but the adsorption of paracetamol on organo-MMT remains less studied. Recently, a micelle-clay complex composed of MMT and octadecyltrimethylammonium was found to be more efficient than activated carbon in removing paracetamol and its biodegradation product from water [42].

The aim of this work was to study the efficiency of removing carbamazepine, ibuprofen, and paracetamol from water using pristine and hydrophobic (trimethyl stearyl ammonium-modified) montmorillonite as adsorbents. The search for new potent adsorbents for these three wide-spread pharmaceuticals is important not only for wastewater decontamination but also for designing detoxification agents to treat medicine overdoses, as was recently proposed in [43].

## 2. Results and Discussion

### 2.1. Characteristics of Clay Minerals and Pharmaceuticals

**Figure 1:**
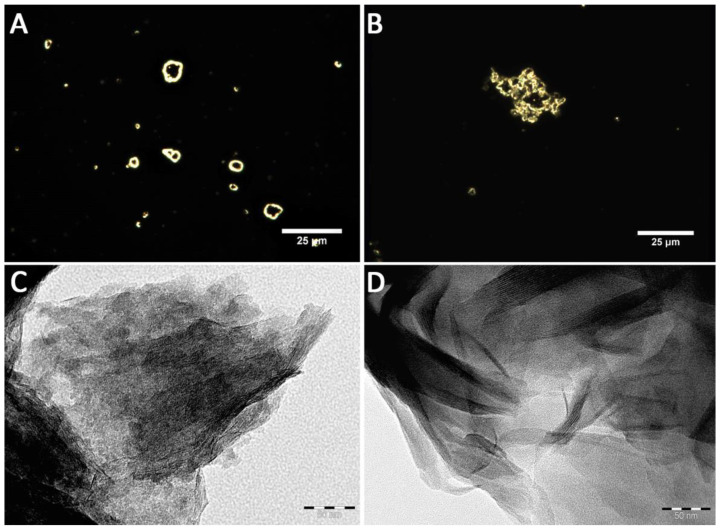
The microscopy images of pristine (**A**,**C**) and hydrophobic (**B**,**D**) montmorillonite obtained using dark-field (**A**,**B**) and transmission electron microscopy (**C**,**D**).

**Figure 2:**
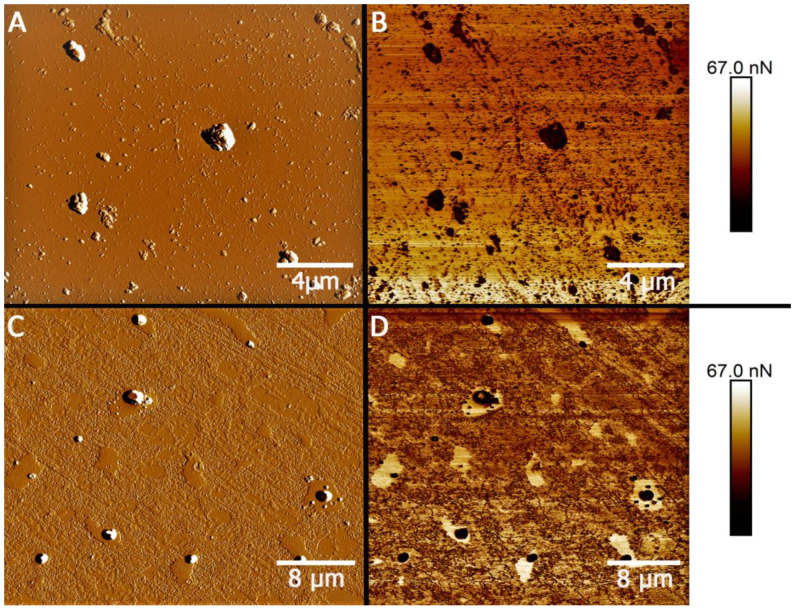
AFM images of pristine (**A**,**B**) and hydrophobic (**C**,**D**) montmorillonite obtained in the Peak Force Error (**A**,**C**) and Adhesion (**B**,**D**) channels.

First, the morphology of pristine and hydrophobic MMT adsorbents was characterized using various microscopic techniques (Figure 1 and Figure 2). MMT modified with trimethyl stearyl ammonium retained the ordered layered structure characteristic of the pristine MMT nanoclay.

Using atomic force microscopy operating in PeakForce Tapping imaging mode, the differences in the adhesion properties of the surface of pristine MMT and that covered with a layer of surfactant were shown. Although the surface topography images of pristine and hydrophobic MMT particles are similar (Figure 2A,C), the changes in nonspecific surface adhesion after MMT hydrophobization can be seen (Figure 2B,D). The surface adhesion values of the studied particles were 5.7 ± 1.4 nN for pristine MMT and 14.9 ± 4.7 nN for hydrophobic one. An increase in the adhesion of clay particles after their hydrophobization was previously observed in case of halloysite modification with octadecyltrimethoxysilane [44]. In Figure 2D, a halo-like feature of highly adhesive material is distinguishable around the hydrophobic MMT particles. This halo presumably belongs to the surfactant used for nanoparticle hydrophobization (trimethylstearylammonium) and has the adhesion (52.6 ± 10.1 nN) almost four times higher than the adhesion of the hydrophobic MMT particles.

The hydrodynamic diameter (D_h_) and zeta potential (ζ) of clay particles were characterized by Dynamic light scattering (DLS) and laser Doppler velocimetry (Table 1). According to the measured zeta potential value, only hydrophobic MMT had a positive charge, which could be beneficial to the adsorption of negatively charged PP.

To study the sorption properties of nanoclays, we used several types of pharmacological preparations, differing in structure and reactivity. Carbamazepine [benzo[b][1]benzazepine-11-carboxamide] is an antiepileptic agent and a normotimic from the group of carboxamide derivatives; Ibuprofen [2-(4-isobutyphenyl) propanoic acid] is a non-steroidal anti-inflammatory drug from the group of propionic acid derivatives, and Paracetamol [N-(4-hydroxyphenyl)acetamide] is analgesic and antipyretic from the group of anilides. These compounds contain a wide range of functional groups: aromatic rings, amide, carbonyl groups, etc., all with potentially different chemical reactivity, which was the reason for the choice of these drugs.

The absorption spectrum of each pharmaceutical was measured, and the peak absorptions all laid in the ultraviolet spectrum at the wavelengths of 285, 220, and 243 nm for carbamazepine, ibuprofen, and paracetamol, respectively.

### 2.2. Effect of Contact Time

In case of reversible adsorption, the PP adsorption on an adsorbent is followed by its partial desorption until the equilibrium of the two processes is reached. To reveal an optimal time for the adsorption process the contact time of the sorbent with PP was varied.

**Figure 3:**
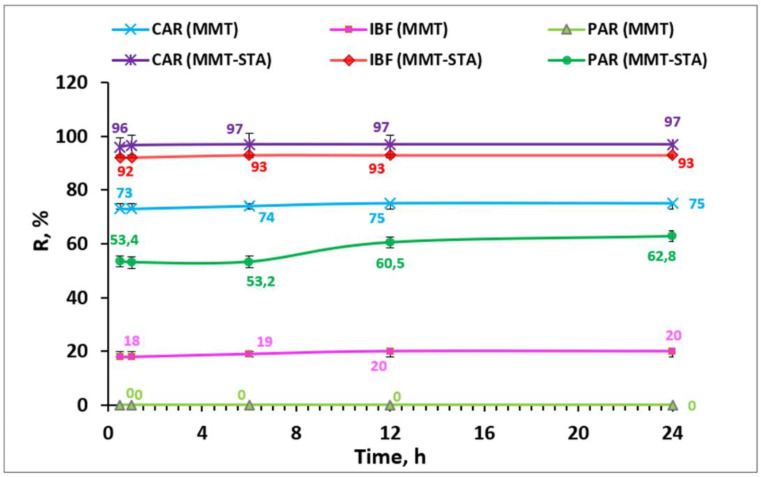
Effect of contact time on the removal efficiency of PP by pristine MMT and modified MMT–STA.

The adsorption proceeded rather quickly (Figure 3), which could be of practical importance for the purification of wastewater from pharmaceuticals. For MMT–STA, the sorption equilibrium was established after 6 h for ibuprofen and carbamazepine and after 24 h for paracetamol with an increase in removal efficiency by 10%. For MMT, the sorption equilibrium was established after 12 h. Thus, the contact time of 24 h was used for obtaining adsorption isotherms.

### 2.3. Adsorption Isotherms

**Figure 4:**
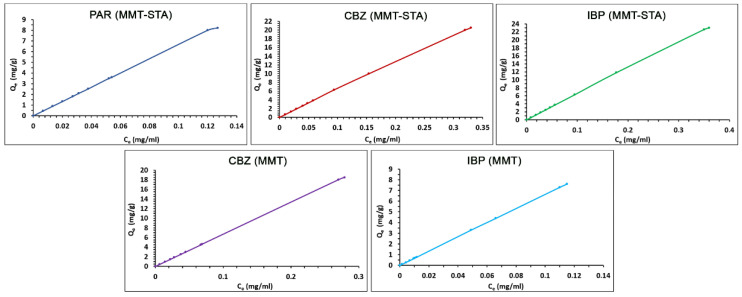
Adsorption isotherm of pharmaceuticals on clays.

The adsorption isotherm describes the relationship between the residual concentration of the adsorbate in the liquid phase and its concentration on the surface of the adsorbent at a certain temperature. The adsorption isotherms of pharmaceuticals by pristine and hydrophobic MMT from water are shown in Figure 4, where C_e_ is the equilibrium concentration of pharmacological preparations in solution and Q_e_ is the amount of adsorbed pharmaceutical per unit adsorbent mass at equilibrium. No isotherm was obtained for paracetamol adsorption by pristine MMT, because no sorption occurred in this case. The sorption isotherms for all other compounds were linear (C-type), according to the classification described by Giles et al. [45].

The linear C-type isotherm corresponds to the constant number of adsorption sites in a wide range of solute concentrations up to the maximum possible adsorption, i.e., as more solute molecules are adsorbed more adsorption sites are created. Such a situation can take place when the solute has a higher attraction for the adsorbate than the solvent [45].

**Figure 5:**
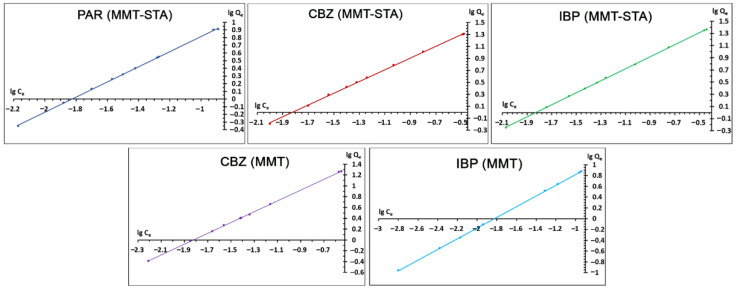
Adsorption of pharmaceuticals onto clay fitted to linearized form of Freundlich isotherm.

The adsorption data were treated using the linearized form of the Freundlich equation (Figure 5). The Freundlich equation is an empirical model describing a multilayer sorption onto the adsorbent surface, characterized by heterogeneous adsorption energies. High linearity of the plot determined by the coefficient of determination (R^2^) points to good correspondence of the adsorption data to the Freundlich model.

The fitting parameters of the model K_f_ and *n* are presented in Table 2. The value of the Freundlich adsorption capacity *n* indicates the favorability of adsorption. The *n* value of 1–2 indicates moderate adsorption capacity, *n* < 1 indicates poor adsorption capacity, and K_f_ between 2 and 10 points to a good adsorption capacity [46]. All K_f_ values obtained were close to 1, meaning moderate sorption capacity of pristine and hydrophobic MMT sorbents towards the pharmaceuticals studied.

### 2.4. Effect of pH

**Figure 6:**
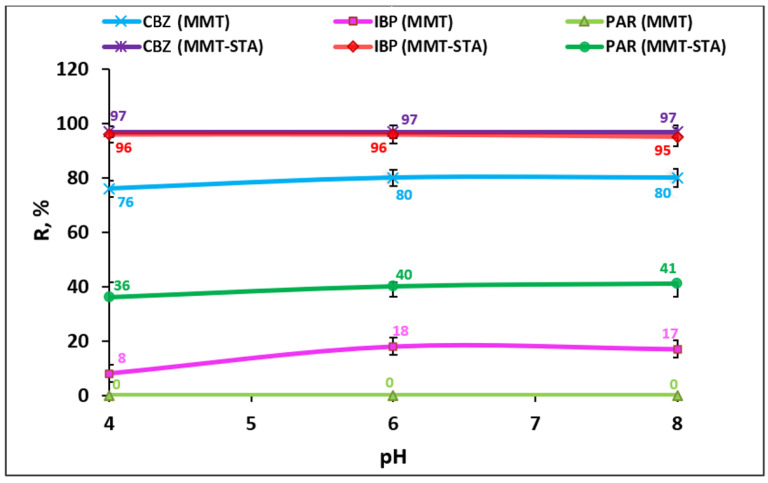
Effect of pH on the removal efficiency of PP by pristine MMT and modified MMT–STA.

The adsorption properties of MMT-STA and MMT at different suspension pH were determined (Figure 6). Figure 6 shows that the pH of the medium in the range from 4 to 8 has no significant effect on the degree of adsorption. The only exception was adsorption of ibuprofen by MMT, with adsorption of IBP decreasing insignificantly by 10% at pH 4. Thus, for practical use, the optimal pH is 6, at which the largest percentage of pharmacological preparations is adsorbed.

The pKa of CBZ and PAR are 13.9 and 9.38, respectively; thus, most of their molecules were uncharged in the studied pH range (pH 4–8). Thus, electrostatic interactions apparently played a little role in the adsorption of these drugs by hydrophobic MMT–STA. According to octanol-water partition coefficients, all the drugs tested were rather hydrophobic, with hydrophobicity decreasing in the row CBZ → IBP→ PAR. Thus, it is more probable that hydrophobic interactions contributed to the efficient adsorption of these drugs by hydrophobic MMT–STA clay. Electrostatic interactions could only take place in case of IPB adsorption on positively charged MMT–STA, which has pKa of 4.91, indicating that most of its molecules would be negatively charged at pH about 4. Thus, the removal efficiency of the three investigated compounds was quite constant in the pH range from 4 to 8. For CBZ adsorption on MMT clay, similar results on pH dependence were reported by other research works [47].

### 2.5. Effect of Temperature

**Figure 7:**
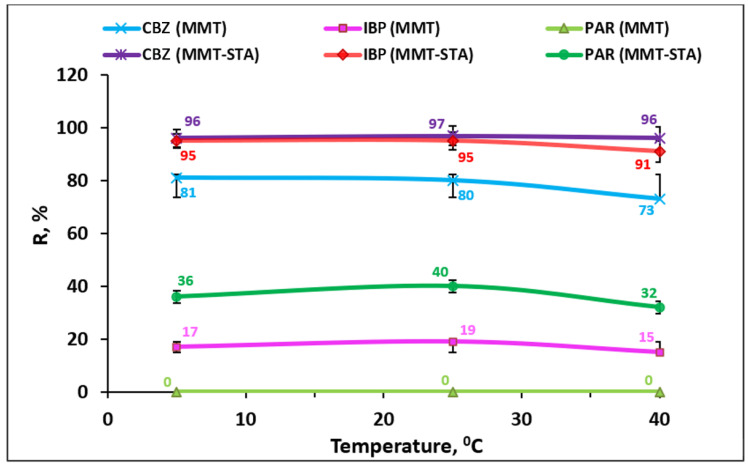
Effect of temperature on the removal efficiency of PP by MMT and modified MMT–STA.

To determine the dependence of the removal efficiency on the temperature, the adsorption properties of clay materials were studied at temperatures of 5 °C, 25 °C, and 40 °C (Figure 7). The temperature had no significant effect on the removal efficiency. The optimal temperature was 25 °C, with adsorption slightly decreasing at higher temperature. The decrease in clay adsorption capacity at higher temperatures can be connected with destabilization of the physical forces involved in the adsorbate–adsorbent interactions [48].

### 2.6. Removal Efficiency

**Figure 8:**
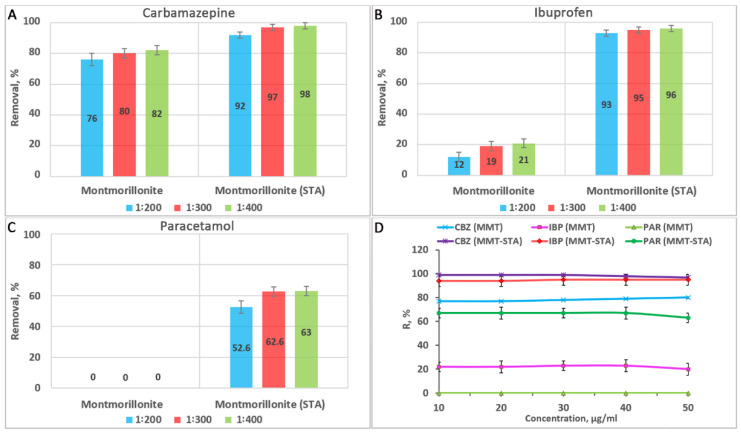
Dependence of the PP removal efficiency on the mass of adsorbents (**A**–**C**) and on the concentration of PP at the mass ratio of clay adsorbent to adsorbate of 300:1 (**D**).

The removal efficiency of PP by MMT and MMT–STA at different masses of adsorbents was studied. Hydrophobic montmorillonite had better adsorption properties compared to pristine montmorillonite (Figure 8). MMT–STA under static conditions with an adsorbate to adsorbent ratio of 1:300 provided almost complete removal (95–97%) of CBZ and IBP in concentrations from 10 to 50 µg/mL (Figure 8D). For PAR in concentrations up to 50 µg/mL, the removal was 63–67%. Under the same conditions, pristine MMT efficiently adsorbed CBZ at concentrations up to 50 µg/mL with the removal rate of 75–80%. IBP was less adsorbed by MMT–STA at concentrations of 10–50 µg/mL, with the removal rate of 16–23%, while MMT did not adsorb PAR at all.

The removal efficiency of natural MMT clay for CBZ obtained in this study was consistent with that observed in [47], which was around 80%. When MMT was used to remove carbamazepine from deionized water and wastewater, the adsorption efficiency was 83.8% and 78.2%, respectively [49]. Noticeably, the concentrations of CBZ studied greatly exceeded those conventionally found in natural wastewaters (up to 6.3 µg/L) [50], therefore untreated MMT clay can be used as an adsorbent for CBZ in wastewater treatment plants. The hydrophobic MMT–STA revealed itself as an even more efficient adsorbent for CBZ, completely absorbing this PP across the whole concentration range studied.

Pristine MMT, in comparison with other clays, kaolinite and goethite, is a better adsorbent for ibuprofen [51]. However, hydrophobisation of MMT can dramatically increase its sorption capacity towards IBP. Thus, the adsorption by MMT-STA was more than four times higher than that by pristine MMT. In other studies, MMT modified with cationic octadecylamine adsorbed up to 99% ibuprofen at a concentration of 0.1–80 mg/L and the adsorption process involved electrostatic and hydrophobic interactions [38,52]. Both hydrophobicity and interlayer spacing of modified MMT clay increases with the increase in the length of the alkyl chain of cation surfactant used for clay modification [53]. Thus, modification of KSF MMT with a shorter chain molecule cetyltrimethylammonium bromide greatly increased the adsorption of IBP compared to non-modified clay, but the maximal removal efficiency reached only 70% [54]. The comparison of IPB sorption on hexadecyltrimethylammonium modified MMT and zeolite revealed that the sorption affinity depended on the adsorbent organic carbon content [55] The better sorption of IBP on hydrophobic MMT compared to pristine MMT can be partly explained by a positive charge of MMT-STA, favouring the adsorption of negatively charged IPB at pH = 6. A lower adsorption capacity of pristine MMT towards IPB compared to MMT–STA is probably associated with the negative charge of the surface of MMT sheets, preventing electrostatic interactions. Anionic drugs can be weakly adsorbed by MMT through other interactions, including hydrogen bonding and Van der Waals forces. 

More efficient sorption of CBZ and IBP by hydrophobic MMT–STA compared to pristine MMT can be understood considering the log K_ow_ values of these compounds which indicate their tendency to distribution into non-water phase. According to log K_ow_, PAR was the most hydrophilic of all the compounds studied and it was less adsorbed by hydrophobic MMT–STA. However, in contrast to pristine MMT, the hydrophobic MMT was capable of PAR sorption, although it demonstrated a tendency to a slight decrease in sorption at the highest PAR concentration. The higher overall efficiency of MMT–STA compared to MMT towards all the compounds studied can also be partly explained by the reported increase in the degree of swelling after MMT modification with trimethyl stearyl ammonium, which increases the ability of the clay to adsorb a significant amount of organic matter [53].

The combination of kinetic studies, X-ray diffraction and molecular dynamics calculations demonstrated that the low adsorption of paracetamol on non-modified smectite clays can be explained by only one adsorption mode possible in this case [56]. The paracetamol molecule adsorbed parallel to the clay surface through van der Waals interactions between the aromatic rings and the clay surface. However, for carbamazepine, an additional adsorption mode was identified, where the interaction between carbamazepine molecule and the clay involved hydrogen bonds and coordination between oxygen atoms and cations. For both paracetamol and carbamazepine, adsorption occurred only on clay external surfaces and neither of the pharmaceuticals entered the interlayer space.

One of the ways to improve the sorption of paracetamol is the use of modified MMT nanoclays. However, in previous studies, montmorillonite modified with titanium oxide exhibited weak sorption properties, adsorbing no more than 20.8% of paracetamol [57]. This value is much lower than that achieved in our study (63–67%) where the hydrophobic MMT-STA was used. However, not all hydrophobizing agents are equally efficient in increasing paracetamol adsorption by MMT. For instance, montmorillonite KSF modified with cetyltrimethylammonium bromide removed maximally 50% of PAR from aqueous solutions [54].

To increase the removal of pharmaceuticals MMT can be mixed with other clays. Thus, palygorskite–montmorillonite was used as a filter material for purifying wastewater from carbamazepine [58]. A natural clay mixture of smectite and kaolinite was used as an effective and inexpensive adsorbent for water purification from ibuprofen and carbamazepine [48].

The application of hydrophobic MMT adsorbents can go beyond the wastewater treatment. In a recent study, hydrophobisation of MMT through modification with di-octadecyl dimethyl ammonium chloride was used to produce Pickering emulsions for the acute oral paracetamol intoxication treatment. In this case, hydrophobic MMT particles both stabilized the detoxifying emulsion and increased the adsorption of paracetamol [43].

## 3. Materials and Methods

### 3.1. Chemical Reagents and Adsorbents

Commercially available montmorillonite Montmorillonite–K 30 (MMT) in the powder form and hydrophobic montmorillonite modified with tertiary ammonium salts (Montmorillonite, containing 25–30 wt% trimethyl stearyl ammonium) (MMT–STA)), in the powder form with a particle size of ≤20 μm were used. The clay minerals were supplied by Sigma-Aldrich, Saint Louis, MO, USA.

Carbamazepine (CBZ), a white crystalline powder with a purity of> 98%, ibuprofen (IBP), a white crystalline powder with a purity of> 98%, and paracetamol (PAR), a white crystalline powder with a purity of 99% were supplied by Sigma-Aldrich, Saint Louis, MO, USA. The physicochemical characteristics and structural formulas of the drugs are given in Table 3 [59].

### 3.2. Characterization of Clay Adsorbents

The morphology of clay materials was characterized using several types of microscopies: transmission electron, atomic force, and dark-field microscopy. For transmission electron microscopy (TEM) investigation [60], a droplet of nanoclay suspension (10 μL) was placed on a copper grid with a carbon substrate and dried at room temperature. Images were taken using a transmission electron microscope (Hitachi HT7700 Exalens, Tokyo, Japan) at 100 kV accelerating voltage. Atomic force microscopy (AFM) images of nanoclays were obtained using a Dimension Icon microscope (Bruker, Billerica, MA, USA), operating in the PeakForce Tapping mode [61]. The samples were dried on a glass substrate at room temperature. The images were obtained using ScanAsyst-air probes (Bruker, Billerica, MA, USA) (nominal length 115 µm, tip radius 2 nm, nominal spring constant 0.4 N m^−1^). The data were processed with the Nanoscope Analysis v.3.0 software (Bruker, Billerica, MA, USA) [62]. The adhesion images were not edited for reliable results. Square areas (300 × 300 nm) on the surface of the particles were used for adhesion calculation. Clay nanoparticles were visualized by dark-field microscopy using an Olympus BX51 (Tokyo, Japan) upright microscope equipped with a CytoViva^®^ enhanced dark field condenser and a DAGE CCD camera (CytoViva, Auburn, AL, USA). The hydrodynamic diameters and zeta potentials of the used nanomaterials were determined using Zetasizer Nano ZS (Malvern Instruments, Malvern, UK).

### 3.3. Adsorption Experiments

Stock solutions (1.0 g/L) of carbamazepine, ibuprofen, and paracetamol were prepared in ethanol and then diluted to desired concentrations using Milli-Q water.

The absorption peak of each pharmaceutical was determined (NanoPhotometer NP80, Implen, Munich, Germany). The calibration curves were plotted in the concentration range of 10–70 µg/mL and used to assess the PP concentration in the supernatant after adsorption. All experiments were performed in triplicate.

To establish the time needed to reach the equilibrium of adsorption, 0.15 g of sorbent and 10 mL of PP solution with a concentration of 50 µg/mL at pH 6 were placed in a flask. The suspensions were sonicated for 30 s. The resulting mixture was thoroughly mixed and kept for 0.5, 1, 6, 12, 24 h at a temperature of 25 °C. Then the solid phase was separated by centrifugation for 15 min at 3500 rpm (CM-6MT, ELMI, Riga, Latvia), and the PP content in the solution was determined by the spectrophotometric method (NanoPhotometer NP80, Implen, Munich, Germany). The removal efficiency by adsorption (R) was calculated according to Equation (1):

(1) $$R=\frac{C_{o}\;-\;C_{e}}{C_{o}}\times 100\%\;$$

where C_o_ is the initial PP concentration in the solution, µg/mL, and C_e_ is equilibrium concentration of PP in solution, µg/mL.

The adsorption of the pharmaceuticals was estimated at different mass ratios of pharmaceutical to adsorbent (1:100–400). For this, 10 mL of PP solution at concentration of 50 μg/mL was added to a flask containing nanoclay (0.05–0.20 g) at pH 6. For better mixing, the suspensions were sonicated for 30 s at 50% amplitude, using a Bandelin SonoPlus ultrasonic probe (Bandelin, Berlin, Germany). The suspensions were stirred on a laboratory stirrer for 24 h at a temperature of 25 °C, then the solid phase was separated by centrifugation for 15 min at 3500 rpm and the PP content in the solution was determined spectrophotometrically

To make an adsorption isotherm, aqueous solutions (10 mL) of drugs were prepared in the concentration range of 10–800 µg/mL. Then, 0.15 g of adsorbent was added to each solution at pH 6 and 25 °C. The suspensions were sonicated (Bandelin SonoPlus, Bandelin, Berlin, Germany) for 30 s, stirred on a laboratory stirrer for 24 h, centrifuged for 15 min at 3500 rpm (CM-6MT, ELMI, Riga, Latvia), and the PP content in the solution was determined by spectrophotometry. The data obtained were used to plot the dependence of the amount of adsorbed solute per unit adsorbent mass Q_e_ (mg/g) on the solute concentration at equilibrium C_e_ (mg/mL). The amount of PP adsorbed per unit clay mass was calculated according to Equation (2):

(2) $$Q_{e}=\frac{\left(C_{o}-C_{e}\right)V}{m}$$

where m is the adsorbent mass and V is solution volume.

The experimental data on adsorption were processed using the Freundlich model, applying Equation (3):

(3) $$Q_{e}\;=\;K_{f}\;\times {\;C}_{e}^{\frac{1}{n}}$$

where Q_e_ is the solute mass adsorbed per unit adsorbent mass at equilibrium (mg/g); C_e_, is the solute concentration in solution at equilibrium (µg/mL); K_f_ is the Freundlich constant, and 1/*n* is the Freundlich exponent indicating the intensity of adsorbent-adsorbate interaction. To find the parameters K_f_ and 1/*n* the linear form of the Freundlich Equation (4) was used:

(4) $$\mathrm{lg}Q_{e}\;=\;\mathrm{lg}K_{f}+\frac{1}{n}\;\mathrm{lg}C_{e}$$

The linearity of the plot indicates the fitness of the adsorption data to the Freundlich model. The parameters K_f_ and 1/*n* were obtained using the intercept and the slope of the plot, respectively.

The dependence of the degree of adsorption on pH and temperature was studied. The required pH value (pH 4–8, t = 25 °C) of solutions was established by adding solutions of hydrochloric acid or sodium hydroxide (0.1 mol/L) and measured using a pH meter SevenCompact S220 (Mettler Toledo, Schwerzenbach, Switzerland). To study the effect of temperature, the adsorption was carried out under static conditions at temperatures of 5 °C, 25 °C, and 40 °C and pH 6. In these experiments, 0.15 g of the adsorbent and 10 mL of a PP solution with a concentration of 50 µg/mL (mass ratio of PP to adsorbent 1:300) were placed in the flask and sonicated for 30 s. The suspensions were stirred on a laboratory stirrer for 24 h, centrifuged for 15 min at 3500 rpm (CM-6MT, ELMI, Riga, Latvia), and the PP content in the supernatant was determined spectrophotometrically (NanoPhotometer NP80, Implen, Munich, Germany).

## 4. Conclusions

Natural sorbents can outperform the synthetic ones due to their significantly higher availability and hence lower production costs. Additionally, synthetic materials not encountered in nature can introduce new environmental pollutants. Nanoclays are natural sorbents the efficiency of which can be increased by chemical modifications to achieve interactions with desired adsorbates. The adsorption properties of natural and hydrophobic montmorillonite were investigated by removing carbamazepine, ibuprofen, and paracetamol from their aqueous solutions. The experimental data on adsorption were best described by the Freundlich isotherm model. Within the pharmaceutical concentration range of 10–50 µg/mL, the optimal mass ratio of adsorbates to adsorbents was 1:300, pH 6, and a temperature of 25 °C. In general, the pH and temperature of the medium did not significantly affect the degree of adsorption of pharmaceuticals, with the adsorption slightly decreasing at 40 °C and pH 4. Comparative analysis of pristine and hydrophobic montmorillonite showed that MMT-STA had betted adsorption properties and removed 95–97% of CBZ or IBP and 63–67% of PAR at concentrations from 10 to 50 μg/mL. For IPB and PAR this was a rather high efficiency in comparison with previous studies, where other modified MMT adsorbents were used. Under the same conditions, pristine montmorillonite effectively adsorbed only CBZ (75–80%), to a lesser extent IBP (16–23%) and did not adsorb PAR. Thus, hydrophobisation significantly increased the sorption properties of MMT, and MMT–STA can be regarded as a promising adsorbent for removing hydrophobic organic contaminants from wastewater. Moreover, the high adsorptive capacity of MMT-STA towards pharmaceuticals studied can find applications not only in wastewater treatment but also for detoxification purposes in case of pharmaceutical overdose.

## Figures and Tables

**Table 1:** The values of the hydrodynamic diameters (D_h_) and zeta potentials (ζ) of the substances used.

Nanomaterials	D_h_, nm	ζ, mV
MMT	1723 ± 65.20	−31.0 ± 1.23
MMT-STA	7002 ± 198.0	20.9 ± 0.25
Carbamazepine	612.0 ± 81.20	−18.9 ± 6.12
Ibuprofen	1950 ± 764.5	−3.64 ± 0.16
Paracetamol	997.2 ± 425.6	−9.76 ± 4.03

MMT = pristine montmorillonite; MMT-STA = hydrophobic montmorillonite.

**Table 2:** Parameters of the Freundlich equation obtained in the analysis of adsorption isotherms of pharmaceuticals.

Fitting Parameters	Adsorption of Pharmaceuticals on Clays				
	PAR (MMT-STA)	CBZ (MMT-STA)	IBP (MMT-STA)	CBZ (MMT)	IBP (MMT)
Freundlich Equation					
K_f_, (mg/g) (mL/mg) ^1/*n*^	63.39	61.99	63.66	66.47	65.07
*n*	1.013	1.017	1.012	0.999	1.007
R^2^	0.99965	0.99952	0.99981	0.99983	0.99991

**Table 3:** Physico-chemical properties of the pharmaceutical preparations studied (M_w_–molecular weight, S_w_–solubility in water, pK_a_–the negative log of the acid dissociation constant, log K_ow_–the octanol/water partition coefficient).

Pharmaceutical Products (PP)	Molecular Formula	Chemical Structure	M_w_ (g.mol ^r−1^)	S_w_ (mg. L^−1^)	pK_a_	log K_ow_
Carbamazepine (CBZ)	C_15_H_12_N_2_O	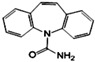	236.3	17.7	13.9	2.45
Ibuprofen (IBP)	C_13_H_18_O_2_	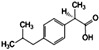	206.3	21.0	4.91	3.97
Paracetamol (PAR)	C_8_H_9_NO_2_	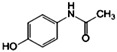	151.2	1.4 × 10^4^	9.38	0.46

## Data Availability

The data that support the findings of this study are available from the corresponding author upon reasonable request.

## References

[1] Evgenidou E.N., Konstantinou I.K., Lambropoulou D.A. (2015). Occurrence and removal of transformation products of PPCPs and illicit drugs in wastewaters: A review. Sci. Total Environ..

[2] Hai F.I., Yang S., Asif M.B., Sencadas V., Shawkat S., Sanderson-Smith M., Gorman J., Xu Z.Q., Yamamoto K. (2018). Carbamazepine as a possible anthropogenic marker in water: Occurrences, toxicological effects, regulations and removal by wastewater treatment technologies. Water.

[3] Macías-García A., García-Sanz-Calcedo J., Carrasco-Amador J.P., Segura-Cruz R. (2019). Adsorption of paracetamol in hospital wastewater through activated carbon filters. Sustainability.

[4] Caban M., Stepnowski P. (2021). How to decrease pharmaceuticals in the environment? A review. Environ. Chem. Lett..

[5] Lewandowski J., Meinikmann K., Krause S. (2020). Groundwater–surface water interactions: Recent advances and interdisciplinary challenges. Water.

[6] Sui Q., Cao X., Lu S., Zhao W., Qiu Z., Yu G. (2015). Occurrence, sources and fate of pharmaceuticals and personal care products in the groundwater: A review. Emerg Contam..

[7] Chander V., Sharma B., Negi V., Aswal R.S., Singh P., Singh R., Dobhal R. (2016). Pharmaceutical compounds in drinking water. J. Xenobiot..

[8] Patra J.K., Das G., Fraceto L.F., Campos E.V.R., Rodriguez-Torres M.D.P., Acosta-Torres L.S., Diaz-Torres L.A., Grillo R., Swamy M.K., Sharma S. (2018). Nano based drug delivery systems: Recent developments and future prospects. J. Nanobiotechnol..

[9] Lopez-Serna R., Petrovic M., Barcelo D. (2012). Occurrence and distribution of multiclass pharmaceuticals and their active metabolites and transformation products in the Ebro River basin (NE Spain). Sci. Total Environ..

[10] Luo Y., Guo W., Ngo H.H., Nghiem L.D., Hai F.I., Zhang J., Liang S., Wang X.C. (2014). A review on the occurrence of micropollutants in the aquatic environment and their fate and removal during wastewater treatment. Sci. Total Environ..

[11] Zhou J.L., Zhang Z.L., Banks E., Grover D., Jiang J.Q. (2009). Pharmaceutical residues in wastewater treatment works effluents and their impact on receiving river water. J. Hazard. Mater..

[12] Vogna D., Marotta R., Andreozzi R., Napolitano A., d’Ischia M. (2004). Kinetic and chemical assessment of the UV/H_2_O_2_ treatment of antiepileptic drug carbamazepine. Chemosphere.

[13] Rivera-Jaimes J.A., Postigo C., Melgoza-Alemán R.M., Aceña J., Barceló D., de Alda M.L. (2018). Study of pharmaceuticals in surface and wastewater from cuernavaca, morelos, mexico: Occurrence and environmental risk assessment. Sci. Total Environ..

[14] Heckmann L.H., Helen A.C., Hooper L., Connon R., Hutchinson T.H., Maund S.J., Sibly R.M. (2007). Chronic toxicity of ibuprofen to *Daphnia magna*: Effects on life history traits and population dynamics. Toxicol. Lett..

[15] Nunes B., Antunes S.C., Santos J., Martins L., Castro B.B. (2014). Toxic potential of paracetamol to freshwater organisms: A headache to environmental regulators?. Ecotoxicol. Environ. Saf..

[16] Jelic A., Gros M., Ginebreda A., Cespedes-Sanchez R., Ventura F., Petrovic M., Barcelo D. (2011). Occurrence, partition and removal of pharmaceuticals in sewage water and sludge during wastewater treatment. Water Res..

[17] Carvalho P.N., Pirra A., Basto M.C.P., Almeida C.M.R. (2013). Activated sludge systems removal efficiency of veterinary pharmaceuticals from slaughterhouse wastewater. Environ. Sci. Pollut. Res..

[18] Radjenovic J., Petrovic M., Barcelo D. (2009). Fate and distribution of pharmaceuticals in wastewater and sewage sludge of the conventional activated sludge (CAS) and advanced membrane bioreactor (MBR) treatment. Water Res..

[19] Martinez F., López-Muñoz M.J., Aguado J., Melero J.A., Arsuaga J., Sotto A., Molina R., Segura Y., Pariente M.I., Revilla A. (2013). Coupling membrane separation and photocatalytic oxidation processes for the degradation of pharmaceutical pollutants. Water Res..

[20] Nariyan E., Aghababaei A., Sillanpää M. (2017). Removal of pharmaceutical from water with an electrocoagulation process; effect of various parameters and studies of isotherm and kinetic. Sep. Purif. Technol..

[21] Jiang M., Yang W., Zhang Z., Yang Z., Wang Y. (2015). Adsorption of three pharmaceuticals on two magnetic ion-exchange resins. J. Environ. Sci..

[22] Klavarioti M., Mantzavinos D., Kassinos D. (2009). Removal of residual pharmaceuticals from aqueous systems by advanced oxidation processes. Environ. Int..

[23] An T., Yang H., Li G., Song W., Cooper W.J., Nie X. (2010). Kinetics and mechanism of advanced oxidation processes (AOPs) in degradation of ciprofloxacin in water. Appl. Catal. B-Environ..

[24] Andrade J.R., Oliveira M.F., da Silva M.G.C., Vieira M.G. (2018). Adsorption of pharmaceuticals from water and wastewater using nonconventional low-cost materials: A review. Ind. Eng. Chem. Res..

[25] Akhtar J., Amin N.A.S., Shahzad K. (2016). A review on removal of pharmaceuticals from water by adsorption. Desalin. Water Treat..

[26] Styszko K., Nosek K., Motak M., Bester K. (2015). Preliminary selection of clay minerals for the removal of pharmaceuticals, bisphenol A and triclosan in acidic and neutral aqueous solutions. Comptes Rendus Chim..

[27] Silva A.R., Cavaleiro A.J., Soares O.S.G.P., Braga C.S.N., Salvador A.F., Pereira M.F.R., Alves M.M., Pereira L. (2021). Detoxification of ciprofloxacin in an anaerobic bioprocess supplemented with magnetic carbon nanotubes: Contribution of adsorption and biodegradation mechanisms. Int. J. Mol. Sci..

[28] Lvov Y., Panchal A., Fu Y., Fakhrullin R., Kryuchkova M., Batasheva S., Stavitskaya A., Glotov A., Vinokurov V. (2019). Interfacial self-assembly in halloysite nanotube composites. Langmuir.

[29] Thiebault T. (2020). Raw and modified clays and clay minerals for the removal of pharmaceutical products from aqueous solutions: State of the art and future perspectives. Crit. Rev. Environ. Sci. Technol..

[30] Thiebault T., Boussafir M., Fougere L., Destandau E., Monnin L., Milbeau C. (2019). Clay minerals for the removal of pharmaceuticals: Initial investigations of their adsorption properties in real wastewater effluents. Environ. Nanotechnol. Monit. Manag..

[31] Zhu R., Chen Q., Zhou Q., Xi Y., Zhu J., He H. (2016). Adsorbents based on montmorillonite for contaminant removal from water: A review. Appl. Clay Sci..

[32] Massaro M., Colletti C.G., Lazzara G., Riela S. (2018). The use of some clay minerals as natural resources for drug carrier applications. J. Funct. Biomater..

[33] Aguzzi C., Cerezo P., Viseras C., Caramella C. (2007). Use of clays as drug delivery systems: Possibilities and limitations. Appl. Clay Sci..

[34] Park J.H., Shin H.J., Kim M.H., Kim J.S., Kang N., Lee J.Y., Kim K.T., Lee J.I., Kim D.D. (2016). Application of montmorillonite in bentonite as a pharmaceutical excipient in drug delivery systems. J. Pharm. Investig..

[35] Pandey S. (2017). A comprehensive review on recent developments in bentonite-based materials used as adsorbents for wastewater treatment. J. Mol. Liq..

[36] Wu Q., Li Z., Hong H., Yin K., Tie L. (2010). Adsorption and intercalation of ciprofloxacin on montmorillonite. Appl. Clay Sci..

[37] Liu N., Wang M.X., Liu M.M., Liu F., Weng L., Koopal L.K., Tan W.F. (2012). Sorption of Tetracycline on Organo-Montmorillonites. J. Hazard. Mater..

[38] Martín J., del Mar Orta M., Medina-Carrasco S., Santos J.L., Aparicio I., Alonso E. (2019). Evaluation of a modified mica and montmorillonite for the adsorption of ibuprofen from aqueous media. Appl. Clay Sci..

[39] Oiwa M., Yamaguchi K., Hayashi H., Saitoh T. (2020). Rapid sorption of fenitrothion on didodecyldimethylammonium bromidemontmorillonite organoclay followed by the degradation into less toxic 3-methyl-4- nitrophenolate. J. Environ. Chem. Eng..

[40] De Oliveira T., Guégan R., Thiebault T., Le Milbeau C., Muller F., Teixeira V., Giovanela M., Boussafir M. (2017). Adsorption of diclofenac onto organoclays: Effects of surfactant and environmental (pH and temperature) conditions. J. Hazard. Mater..

[41] Zhang W., Ding Y., Boyd S.A., Teppen B.J., Li H. (2010). Sorption and desorption of carbamazepine from water by smectite clays. Chemosphere.

[42] Karaman R., Khamis M., Abbadi J., Amro A., Qurie M., Ayyad I., Ayyash F., Hamarsheh O., Yaqmour R., Nir S. (2016). Paracetamol biodegradation by activated sludge and photocatalysis and its removal by a micelle–clay complex, activated charcoal, and reverse osmosis membranes. Environ. Technol..

[43] Zhang W., Li M., Fan X., Sun X., He G. (2018). Preparation and in vitro evaluation of hydrophobic-modified montmorillonite stabilized pickering emulsion for overdose acetaminophen removal. Can. J. Chem. Eng..

[44] Rozhina E., Panchal A., Akhatova F., Lvov Y., Fakhrullin R. (2020). Cytocompatibility and cellular uptake of alkylsilane-modified hydrophobic halloysite nanotubes. Appl. Clay Sci..

[45] Giles C.H., MacEwan T.H., Nakhwa S.N., Smith D. (1960). Studies in adsorption. Part XI. A system of classification of solution adsorption isotherms, and its use in diagnosis of adsorption mechanisms and in measurement of specific surface areas of solids. J. Chem. Soc..

[46] Brdar M., Šćiban M., Takači A., Došenović T. (2012). Comparison of two and three parameters adsorption isotherm for Cr (VI) onto Kraft lignin. Chem. Eng. J..

[47] Mahouachi L., Rastogi T., Palm W.U., Ghorbel-Abid I., Chehimi D.B., Kümmerer K. (2020). Natural clay as a sorbent to remove pharmaceutical micropollutants from wastewater. Chemosphere.

[48] Khazri H., Ghorbel-Abid I., Kalfat R., Trabelsi-Ayadi M. (2017). Removal of ibuprofen, naproxen and carbamazepine in aqueous solution onto natural clay: Equilibrium, kinetics, and thermodynamic study. Appl. Water Sci..

[49] Alaghmand M., Alizadeh-Saei J., Barkat S. (2020). Adsorption and removal of a selected emerging contaminant, carbamazepine, using humic acid, humasorb and montmorillonite. equilibrium isotherms, kinetics and effect of the water matrix. J. Environ. Sci. Health A.

[50] Ternes T.A. (1998). Occurrence of drugs in German sewage treatment plants and rivers. Water Res..

[51] Behera S.K., Oh S.Y., Park H.S. (2012). Sorptive removal of ibuprofen from water using selected soil minerals and activated carbon. Int. J. Environ. Sci. Technol..

[52] Malvar J.L., Martín J., del Mar Orta M., Medina-Carrasco S., Santos J.L., Aparicio I., Alonso E. (2020). Simultaneous and individual adsorption of ibuprofen metabolites by a modified montmorillonite. Appl. Clay Sci..

[53] Yang Z., Wang W., Tai X., Wang G. (2019). Preparation of modified montmorillonite with different quaternary ammonium salts and application in Pickering emulsion. New J. Chem..

[54] Vallova S., Plevova E., Smutna K., Sokolova B., Vaculikova L., Valovicova V., Hundakova M., Praus P. Removal of analgesics from aqueous solutions onto montmorillonite KSF. J. Therm. Anal. Calorim..

[55] Choi J., Shin W.S. (2020). Removal of salicylic and ibuprofen by hexadecyltrimethylammonium-modified montmorillonite and zeolite. Minerals.

[56] Corbin G., Vulliet E., Lanson B., Rimola A., Mignon P. (2021). Adsorption of pharmaceuticals onto smectite clay minerals: A combined experimental and theoretical study. Minerals.

[57] Chauhan M., Saini V.K., Suthar S. (2020). Ti-pillared montmorillonite clay for adsorptive removal of amoxicillin, imipramine, diclofenac-sodium, and paracetamol from water. J. Hazard. Mater..

[58] Berhane T.M., Levy J., Krekeler M.P.S., Danielson N.D., Stalcup A. (2015). Sorption–desorption of carbamazepine bypalygorskite–montmorillonite (PM) filter medium. J. Hazard. Mater..

[59] Trenholm R.A., Vanderford B.J., Holady J.C., Rexing D.J., Snyder S.A. (2006). Broad range analysis of endocrine disruptors and pharmaceuticals using gas chromatography and liquid chromatography tandem mass spectrometry. Chemosphere.

[60] Dzamukova M.R., Naumenko E.A., Lannik N.I., Fakhrullin R.F. (2013). Surface-modified magnetic human cells for scaffold-free tissue engineering. Biomater. Sci..

[61] Kryuchkova M., Fakhrullin R. (2018). Kaolin alleviates graphene oxide toxicity. Environ. Sci. Technol. Lett..

[62] Akhatova F., Fakhrullina G., Khakimova E., Fakhrullin R. (2018). Atomic force microscopy for imaging and nanomechanical characterisation of live nematode epicuticle: A comparative *Caenorhabditis elegans* and *Turbatrix aceti* study. Ultramicroscopy.

